# Multimodal Prehabilitation in Heart Transplant Recipients Improves Short-Term Post-Transplant Outcomes without Increasing Costs

**DOI:** 10.3390/jcm12113724

**Published:** 2023-05-28

**Authors:** Manuel López-Baamonde, María José Arguis, Ricard Navarro-Ripoll, Elena Gimeno-Santos, Bárbara Romano-Andrioni, Marina Sisó, Silvia Terès-Bellès, Antonio López-Hernández, Adrià Burniol-García, Marta Farrero, Raquel Sebio-García, Elena Sandoval, María Sanz-de la Garza, Julián Librero, Ana García-Álvarez, María Ángeles Castel, Graciela Martínez-Pallí

**Affiliations:** 1 Anesthesiology and Intensive Care Department, Hospital Clínic, 08036 Barcelona, Spain; 2 Prehabilitation Group (Surgifit), Hospital Clínic, 08036 Barcelona, Spain; 3 Institut d’Investigacions Biomèdiques August Pi i Sunyer (IDIBAPS), University of Barcelona (UB), 08193 Barcelona, Spain; 4 Barcelona Institute for Global Health (ISGlobal), 08036 Barcelona, Spain; 5 Endocrinology and Nutrition Department, Hospital Clínic, 08036 Barcelona, Spain; 6 Institut Clínic de Malalties Digestives i Metabòliques, Hospital Clínic de Barcelona, 08036 Barcelona, Spain; 7 Econometrics Department, Universitat Pompeu Fabra, 08002 Barcelona, Spain; 8 Heart Failure and Heart Transplantation Unit, Cardiology Department, Hospital Clínic Barcelona, 08036 Barcelona, Spain; 9 Cardiology Department, Cardiovascular Institute, Hospital Clínic de Barcelona—IDIBAPS, 08036 Barcelona, Spain; 10 Physical Medicine and Rehabilitation Department, Hospital Clínic de Barcelona, 08036 Barcelona, Spain; 11 Cardiovascular Surgery Department, Hospital Clínic de Barcelona, 08036 Barcelona, Spain; 12 Navarra Institute for Health Research (IdiSNA), 31008 Pamplona, Spain; 13 Navarrabiomed, Hospital Universitario de Navarra (HUN), Universidad Pública de Navarra (UPNA), 31008 Pamplona, Spain; 14 Red de Investigación en Servicios Sanitarios y Enfermedades Crónicas (REDISSEC), 28029 Madrid, Spain; 15 CIBER-CV, Centro de Investigación Biomédica en Red Enfermedades Cardiovasculares, Instituto de Salud Carlos III, 28029 Madrid, Spain; 16 Biomedical Research Networking Center on Respiratory Diseases (CIBERES), 28029 Madrid, Spain

**Keywords:** heart transplantation, prehabilitation, preoperative optimization, aerobic capacity, postoperative complications, cost-analysis

## Abstract

(1) Background and aim: This study aimed to investigate the impact of prehabilitation on the postoperative outcomes of heart transplantation and its cost-effectiveness. (2) Methods: This single-center, ambispective cohort study included forty-six candidates for elective heart transplantation from 2017 to 2021 attending a multimodal prehabilitation program consisting of supervised exercise training, physical activity promotion, nutritional optimization, and psychological support. The postoperative course was compared to a control cohort consisting of patients transplanted from 2014 to 2017 and those contemporaneously not involved in prehabilitation. (3) Results: A significant improvement was observed in preoperative functional capacity (endurance time 281 vs. 728 s, *p* < 0.001) and quality-of-life (Minnesota score 58 vs. 47, *p =* 0.046) after the program. No exercise-related events were registered. The prehabilitation cohort showed a lower rate and severity of postoperative complications (comprehensive complication index 37 vs. 31, *p =* 0.033), lower mechanical ventilation time (37 vs. 20 h, *p =* 0.032), ICU stay (7 vs. 5 days, *p =* 0.01), total hospitalization stay (23 vs. 18 days, *p =* 0.008) and less need for transfer to nursing/rehabilitation facilities after hospital discharge (31% vs. 3%, *p =* 0.009). A cost-consequence analysis showed that prehabilitation did not increase the total surgical process costs. (4) Conclusions: Multimodal prehabilitation before heart transplantation has benefits on short-term postoperative outcomes potentially attributable to enhancement of physical status, without cost-increasing.

## 1. Introduction

Heart transplantation is currently the gold-standard therapy for selected patients with advanced refractory heart failure [1,2]. However, heart transplantation requires an aggressive surgery and represents enormous physiological stress for the patient with both immediate and long-term consequences [3,4].

Frailty is particularly prevalent among heart transplantation candidates; it represents a status of global physical dysfunction characterized by limited aerobic capacity, reduced exercise tolerance, and in advanced stages, malnutrition, and sarcopenia [5,6,7]. This physical deconditioning generates a vicious circle leading to avoidance of physical activity which, in turn, further worsens functional capacity leading to impaired quality of life [5,8,9]. This situation may progress while patients are on the waiting list as donor heart availability is limited and waiting times can be long. Consequently, patients undergo heart transplantation with a very poor functional, nutritional, and emotional status, which negatively contributes to morbidity and mortality after heart transplantation [9,10,11]. In advanced cases, this situation may even preclude heart transplantation. Moreover, frailty is one of the strongest predictors of increased post-transplant mortality and is associated with a higher number of complications, prolonged hospitalizations, and higher health-resources consumption [12].

Multimodal prehabilitation has emerged in recent years as an innovative intervention that focuses on optimizing physiological and psychological resilience to withstand the upcoming stress of surgery. It involves a comprehensive, short-term, patient-centered program designed to improve the patient’s physical function, nutritional and psychological status [13,14], and to optimize the management of existing comorbidities [15], eventually aiming at decreasing the incidence and severity of postoperative complications and enhancing recovery after surgery. Over the last few years, prehabilitation programs have shown efficacy to prevent postoperative complications in selected high-risk surgical populations [16,17,18,19]. Time spent on the waiting list prior to heart transplantation provides an opportunity to optimize the recipient’s condition reducing preoperative risk factors. Intuitively, this bundle of care appears to be ideally suited to counterbalance the clinical deterioration and poor functional capacity of these patients [17,20].

Guidelines and Scientific Societies recommend exercise training for heart failure patients as a part of their treatment to both prevent and reverse frailty [21,22,23], preventing the heart failure syndrome-related progressive physical decline [24,25,26]. However, for patients with advanced heart failure, especially those on the heart transplantation waiting list, there is currently limited data available, likely because they are frequently considered overly feeble to train. The fear of complications during the exercise and the need for monitoring and personalized training by experienced personnel complicates its implementation as a part of their standard therapy. Moreover, the costs of establishing a prehabilitation program might be considered an economic burden requiring extra resources.

In this sense, our group recently showed in a pilot study the feasibility and efficacy of prehabilitation in heart transplantation candidates for improving functional capacity and quality of life [27]. Encouraged by these results, we designed the present study aiming to investigate the impact of a personalized multimodal prehabilitation intervention in heart transplantation candidates to minimize both pre and postoperative morbidity and enhance recovery. Secondly, we performed a cost analysis of the program to test the hypothesis that a prehabilitation program in heart transplantation candidates reduces hospitalization costs and is cost-effective.

## 2. Materials and Methods

### 2.1. Study Design

A single-center, ambispective cohort study was designed involving consecutive elective heart transplantation candidates from July 2017 to July 2021 once officially included in the waiting list. The trial obtained local ethical committee approval (HCB/2017/0708) and was registered on ClinicalTrials.gov (NCT03466606). Written consent was obtained for all patients participating in the prehabilitation group.

### 2.2. Participants

From July 2017, all patients included in the waiting list for elective heart transplantation were considered for inclusion in the prehabilitation program. Exclusion criteria were clinical instability precluding exercise training, refusal, or unavailability to participate. Exercise training sessions were delivered at the outpatient clinic and patients’ agreement to attend twice weekly for at least eight weeks (intensive phase) was considered a mandatory requirement.

The control group consisted of a historical cohort of 39 consecutive elective heart transplantation recipients from 2014 to 2017 (prior to the implementation of the prehabilitation program on July 2017), and 12 contemporaneous elective heart transplantation recipients who were not involved in the prehabilitation program due to logistic issues (waiting-list period <2 weeks or not being able to attend twice a week). Data from those patients were obtained from the transplant database and hospital medical records.

### 2.3. Intervention

A baseline assessment of prehabilitation patients was performed during the first week after being included in the heart transplantation waiting list and all participants were reassessed eight weeks thereafter, once the intensive training phase was completed.

The assessment consisted of (i) clinical history and physical examination; (ii) Clinical Frailty Scale (CFS) [28]; (iii) forced spirometry test (BodyBox Plethysmography; Medisoft; Sorinnes, Belgium); (iv) functional capacity evaluation by standard incremental cycle ergometer cardiopulmonary exercise testing (CPET) and endurance time (ET) measured by a cycling constant work-rate exercise testing at 80% of peak oxygen uptake (Ergocard Professional; Medisoft; Sorinnes, Belgium), 6-Minute Walking Test (6MWT), hand-grip strength, and 30” Sit-To-Stand (STS) test; (v) physical activity by the Yale Physical Activity Survey (YPAS); (vi) health-related quality of life by Minnesota Living with Heart Failure Questionnaire (MLHFQ); (vii) emotional status by Hospital Anxiety and Depression Scale (HADS); and (viii) nutritional status by Patient-Generated Subjective Global Assessment, a 3-day food record, and nutritional profile determined by blood sample analysis.

### 2.4. Prehabilitation Program

The intervention was designed to improve (i) functional capacity by exercise training and promotion of physical activity, (ii) nutritional status by nutritional counseling and whey protein supplementation, and (iii) psychological resilience using mindfulness therapy.

The physical program included three main actions: (i) a motivational interview, (ii) a moderate to high-intensity exercise training program, and (iii) promotion of daily physical activity. The exercise training consisted of one-hour sessions of individualized, supervised moderate to high-intensity interval training (HIIT) and resistance training at the hospital outpatient gym facility twice weekly for eight weeks. A sports cardiologist performed the exercise training prescription, and the sessions were conducted by a physical therapist.

HIIT was performed on a stationary bicycle (Bike Forma; Technogym; Cesena, Italy). The program was personalized to subjects according to their peak work rate (WR) performed on CPET at baseline assessment. Each session included 5 min of warm-up and 5 min of cool-down pedaling at 30–40% of the peak WR. The interval training consisted of at least five rounds combining 2 min of high-intensity exercise (starting at 70% of peak WR and progressing to 90–100% of peak WR through the program) interspersed with 3 min of low-intensity recovery periods (40–50% of the peak WR). WR progress during the sessions was tailored on an individual basis, according to the subjects’ symptoms and response to the exercise in previous sessions, to maximize the training effect. All subjects were monitored during the HIIT using a 3-lead electrocardiogram, pulse-oximetry, non-invasive arterial pressure, and levels of self-perceived exertion using the modified Borg scale. Strength training was performed (if not contraindicated) and consisted of upper-limb and core muscle exercise based on local muscular exhaustion within the range of 6 to 12 repetitions and avoiding Valsalva maneuvers. The intensity and/or the number of repetitions increased every week when symptomatology allowed it. In addition, all patients were instructed on breathing exercises with an incentive inspirometer (Coach 2; Smith Medical; London, UK).

After completing the first eight weeks and until heart transplantation, patients followed a mixed maintenance program consisting of one session per week of supervised exercise training and were encouraged to maintain a physical activity plan using community-based facilities or home-based exercising.

The nutritional intervention included nutritional education and a tailored dietary plan according to clinical nutrition in surgery ESPEN guidelines [29] based on the Mediterranean diet. Moreover, participants were prescribed dietetic recommendations to enhance protein intake including whey protein supplementation (Fresubin^®^ protein powder, Fresenius-Kabi, Madrid, Spain) within 1 h after exercise to maximize muscle protein synthesis [30], and before going to sleep to achieve an intake of 1.5–2 g/kg/day of protein. These recommendations were prescribed to all patients if not contraindicated and individualized advice was given if any other supplementation was needed.

All patients were invited to attend a weekly mindfulness group session. This anxiety-coping intervention was strongly recommended to those patients showing signs of anxiety/depression (defined by a HADS score >8). A weekly 60-min group session of breathing and relaxation exercises was conducted by a mindfulness-based stress reduction expert psychologist.

Usual care for both groups consisted of regular cardiological follow-up with medical and heart failure nurse visits, physical activity recommendations, intravenous iron administration if iron deficiency anemia, and nutritional intervention if needed.

### 2.5. Costs

The analysis included heart transplantation surgical procedures, direct hospitalization (until discharge), and prehabilitation costs. Data were obtained through micro-costing techniques according to resource use, combined with diagnostic-related center-specific hospital fees. Prehabilitation costs included specialists’ fees (physical therapist, nutritionist, and psychologist), gym structural costs (hospital-specific fee), and protein costs.

### 2.6. Outcomes

Predefined main study outcome variables assessment was blinded to the interventional groups and included: in-hospital complications according to Clavien-Dindo Classification [31,32] and Comprehensive Complication Index (CCI) [33], postoperative mechanical ventilation time, intensive care unit (ICU) length-of-stay and total hospitalization stay, destination at hospital discharge (home vs. rehabilitation facility), and hospital readmissions during the first 30 days as well as mortality at 30 days, 3 months, and 1 year. To minimize variability, it is important to note that decisions about ventilation time, ICU length of stay, and total length of stay as well as the discharge from ICU to a normal ward, and the destination at hospital discharge follow standardized procedures according to the center protocol.

### 2.7. Statistical Analysis and Sample Size Estimation

Study data were exhaustively collected and managed using Research Electronic Data Capture (REDCap) tools [34,35].

Considering CCI as the primary outcome and assuming a pooled standard deviation of 20 units, the study would require at least a sample size of 28 for each group to achieve a power of 80% and a level of significance of 5% (two-sided), for detecting a true difference in means between the test and the reference group of −15 (from 50 to 35) units [36].

Continuous variables are described by mean (standard deviation) or median (interquartile range (Q1–Q3)) as appropriate, while categorical variables are presented as frequencies (percentages). Costs are described by median (interquartile range (IQR)), and the difference between control and intervention (prehabilitation) groups, so positive values should be interpreted as savings.

The normality of distribution was assessed with the Shapiro-Wilk Test. Between-group comparison of continuous variables and costs was performed using either Student’s *t*-test or the Mann–Whitney U test according to their distribution while Pearson’s χ^2^ test or Fischer’s test was used for categorical variables. Quantile regression was used for medians. To control for the usually skewed distribution of costs, a bootstrapping analysis was performed to increase the robustness of the analysis.

All comparisons were two-sided, with a significance level of 0.05. All statistical analyses were made with R version 4.0.2 [37], (R-Foundation, Vienna, Austria) software or STATA v.17 software [38].

## 3. Results

**Figure 1:**
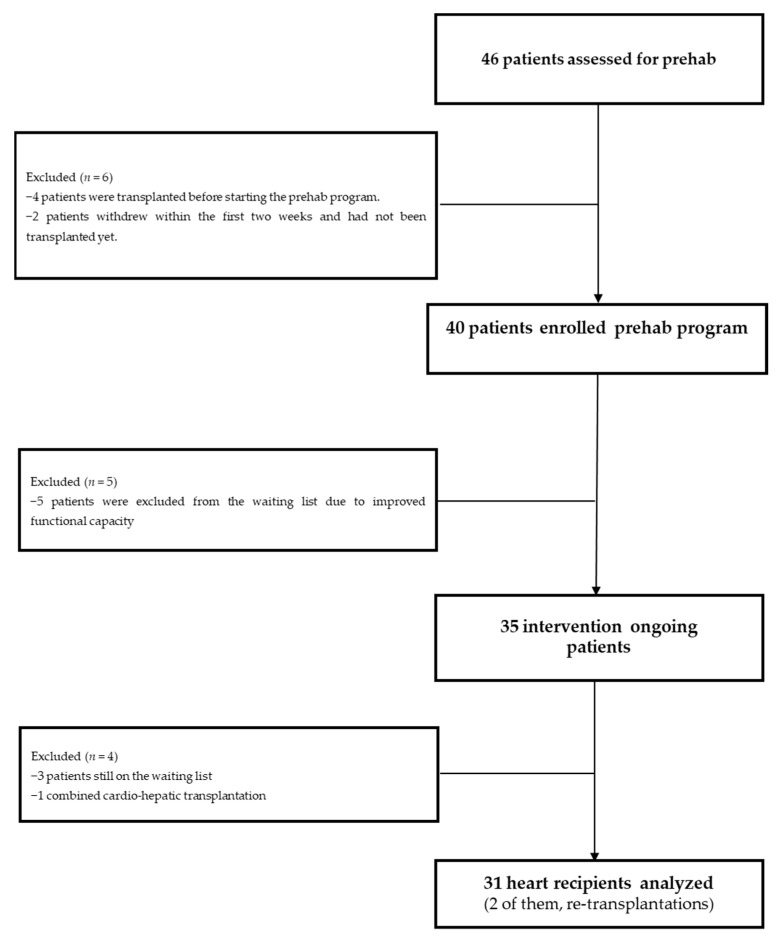
Flowchart.

Between July 2017 and July 2021, 46 heart transplantation candidates were invited to participate in the prehabilitation program as displayed in the study flowchart (Figure 1). All of them gave consent and underwent baseline assessments (Table S1, Supplementary Materials).

However, four patients underwent transplantation before starting the program and were analyzed as part of the control group and two voluntarily abandoned the program within the first week. It is important to note that none of these four patients was prioritized due to clinical need, and we also performed the analysis with them and without them and the results did not change, thus we decided to maintain them in the control group. Five heart transplantation candidates were eventually removed from the waiting list due to significant improvement in their functional capacity after the prehabilitation program intensive phase; five patients were still on the waiting list at the time of performing the analysis (including those two ones who voluntarily abandoned within the first week) and one last patient was excluded because he received a combined cardio-hepatic transplant. Thus, from the initial sample of 46 patients, 31 completed the intensive phase of the prehabilitation program and were transplanted during the study period. These 31 patients were compared to a control group of consecutive 51 heart transplantation recipients as described before.

The demographics and baseline clinical characteristics of both groups are summarized in Table 1. Patients’ characteristics were balanced between study groups including etiology of heart failure, presence of moderate to severe pulmonary hypertension, and use of levosimendan, or INTERMACS classification at the time of heart transplantation, among others. Of note, pulmonary vascular resistance was higher in the prehabilitation group (1.9 ± 1 vs. 2.6 ± 2, *p* = 0.014) and 42% of heart transplantation candidates in this group received intermittent inotropic treatment with levosimendan. Interestingly, prehabilitation group showed a significant trend for lower INTERMACS classification at last month’s previous HT. The median time on the waiting list was longer in the prehabilitation group compared to the control group (186 (93–368) vs. 100 (28–277) days, *p =* 0.016).

### 3.1. Preoperative Effects of Prehabilitation

The median duration of the program was 14 (8–22) weeks and during this time patients attended a median of 25 (13–33) supervised training sessions. Overall, patients attended a mean of 81% (18) of the planned sessions. No cardiovascular or other exercise-related adverse events were registered during the training. All patients received the nutritional intervention, and 22 patients attended a median of 3 (1–8) mindfulness sessions.

Twenty-four patients were re-assessed eight weeks after starting the prehabilitation program as they remained on the waiting list (Table 2). The other seven patients underwent heart transplantation prior to the scheduled reassessment at eight weeks. Patients showed an improvement in functional capacity measured by CPET (ET, from 281 (208, 380) to 728 (397, 900) seconds, *p* < 0.001, and peak oxygen uptake (VO_2_max), from 10.1 (8, 13) to 12.5 (10, 14.78) mL/kg/min, *p =* 0.034) as well as physical activity levels (YPAS, from 24 (15, 37) to 49 (38, 60), *p* < 0.001) and quality of life (MLHFQ from 58 ± 19 to 47 ± 19, *p* = 0.046) compared to baseline measurements (Table 2).

### 3.2. Impact of the Intervention on Postoperative Outcomes

**Figure 2:**
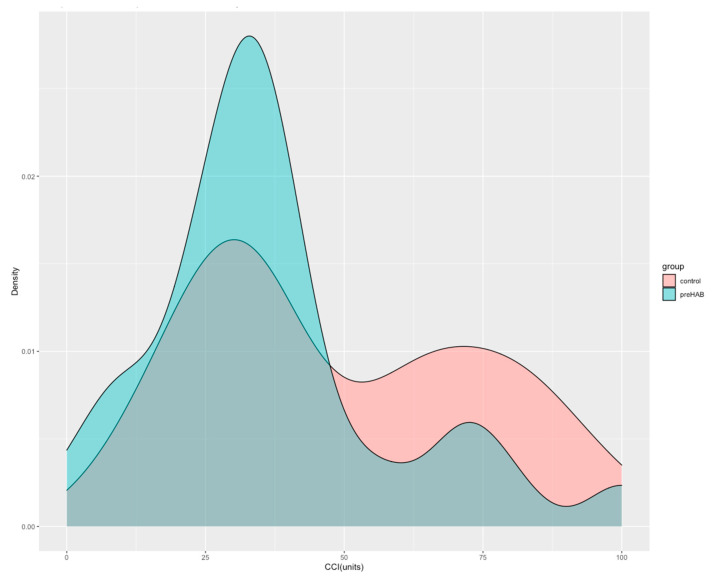
Probability density distribution of the Comprehensive Complication Index according to prehabilitation intervention. The figure shows the probability density function (PDF) of the CCI score according to prehabilitation intervention. The integral over the entire PDF space (area under the curve) is equal to 1. It can be interpreted as providing a relative likelihood that a person from each group would be close to that CCI unit.

At hospital discharge, the rate of postoperative complications per patient was lower in patients undergoing prehabilitation compared to controls (3 vs. 5, *p =* <0.001) attributable to fewer medical complications (2 vs. 5, *p* < 0.001). Patients attending prehabilitation also experienced lower severity of total complications (CCI 31 (23–41 vs. 37 (30–2), *p =* 0.033) (Figure 2) (Table 3).

When analyzing the disaggregated complications, the intervention group showed a lower rate of severe patient myopathy requiring intensified rehabilitation (2 (7%) vs. 15 (29%), *p =* 0.027), paralytic ileus (0 (0%) vs. 10 (19.6%), *p =* 0.022), and rate of arrhythmia requiring antiarrhythmic drugs (1 (3%) vs. 15 (29%), *p =* 0.009) (Table 4).

Moreover, the intervention group required less time for mechanical ventilation after heart transplantation surgery (20 (12–52) vs. 37 (12–143) hours, *p =* 0.03), had lower ICU length of stay (5 (3–7) vs. 7 (5–14) days, *p =* 0.01), as well as reduced total hospitalization length of stay (18 (16–22) vs. 23 (18–38) days, *p =* 0.008). Almost all patients in the prehabilitation group were discharged home (94%), whereas, in the control group, 31.4% of them were required to be transferred to nursing/rehabilitation facilities (*p =* 0.009) after hospital discharge (Table 3). There were no differences in primary graft failure, 30-day re-hospitalization after discharge, or in-hospital 30-day, 3-month, and 1-year mortality between groups.

### 3.3. Costs and Economic Impact

The median cost per patient of the prehabilitation program was 2032€ (1393–3480) (mainly driven by supervised exercise training (1670 € (1020–3154)). The healthcare-related median cost for the HT index hospitalization, including the cost of the prehabilitation program, did not show differences between groups (prehabilitation group: 49,770 € (44,999–54,432) vs. control group: 54,748 € (45,765–79,777); *p* = 0.254) (Table 5).

**Figure 3:**
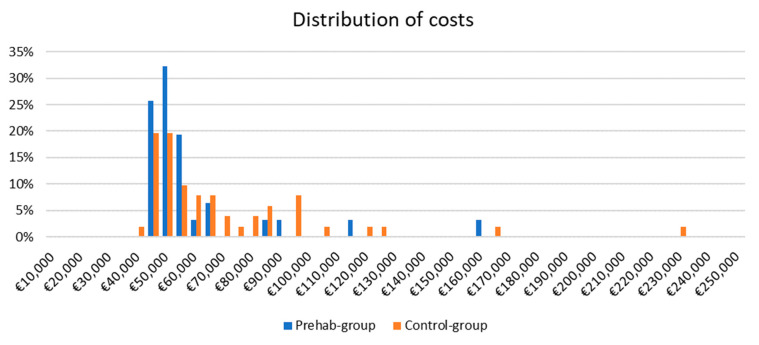
Distribution of costs between groups. The vertical axis shows the proportion of observations in each cost level.

**Figure 4:**
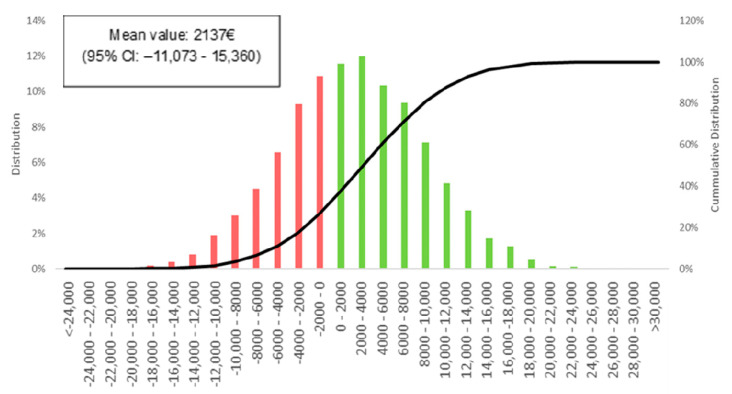
Distribution of bootstrapping results. Red columns represent the iterations where the prehabilitation group had higher costs, and green columns represent those in which the prehabilitation group had lower costs. The black line represents the cumulative distribution of the results, we can see how red columns represent only 38% of iterations. CI: Confidence interval.

Both study groups showed a marked skewness in the distribution of costs (Figure 3). To provide a robust analysis a bootstrapping approach (10,000 iterations) was performed to calculate the means and 95% CI of the difference in per-patient costs between the two groups (Figure 4). The difference in costs was non-statistically significant (2137 € 95% CI: −11,073–15,360), however, over 60% of iterations showed smaller costs for the prehab-group (Figure 4). This difference was presumably driven by the reduction in postoperative complications, the ICU and total length of stay, and pharmacy and blood costs.

## 4. Discussion

Our main findings support the beneficial impact of a multimodal prehabilitation intervention in the short-term postoperative outcome of heart transplantation recipients without increasing direct healthcare costs, which may be interpreted as evidence of higher value for money (cost-effective intervention).

While cardiopulmonary rehabilitation programs have become highly standardized for cardiac patients after an event or a major health episode [22], prehabilitation is a novel concept that proposes physical and psychological training as a preparatory intervention prior to a scheduled surgery/therapy aiming to optimize/improve risk profile [17,20,39]. Since there is a strong relationship between the preoperative functional status (measured as aerobic capacity, frailty, physical activity, etc.) and postoperative outcome [9,10], the rationale for prehabilitation implementation in the heart transplantation setting is appropriate and seems desirable. For instance, in our study, more than 40% of heart transplantation candidates were considered frail (CFS > 5). Benefits of prehabilitation have been already demonstrated in other surgical patients such as cardiac revascularization [40], colorectal [41,42], lung resection [43], vascular and abdominal aorta aneurysm [17,44], major abdominal [16,45,46], and lumbar fusion surgeries [47]. There is also an increasingly reported experience showing the benefit of prehabilitation in patients before other solid organ transplantations [48,49]. Exercise training has been shown to be effective in improving fitness conditions, functional capacity, and quality of life in patients suffering from heart failure [50,51]. Hence, even in the absence of solid evidence, guidelines have been advocating for multimodal preoperative approaches to optimize heart transplantation outcomes and cardiac surgery candidates [52,53,54]

Consistently, we had already reported the feasibility and efficacy of prehabilitation on the enhancement of physical status in patients waiting for heart transplantation [27], According to our previous experience [27], aerobic capacity measured by CPET substantially improved after the 8-week intensive phase of the program. In addition, the level of physical activity (YPAS) and quality of life (MLHFQ) improved as well. Thus, our data support the claim that a prehabilitation program can prevent the clinical deterioration of heart transplantation candidates while on the waiting list. Indeed, it is worth noting that five patients improved their functional capacity parameters enough to be withdrawn from the waiting list. Considering that long-term survival after heart transplantation is limited (10-year survival surrounding 53% [55], and median survival, although progressively improving, scarcely exceeds 12 years) [11,56], delaying the time until transplantation may result in increased overall survival.

More importantly, the current study shows that a prehabilitation program prior to heart transplantation improves postoperative outcomes by reducing postoperative complications (lower rate of medical complications) and their severity (lower CCI) compared to the control cohort. CCI, which has been recently validated in cardiac surgery [57], summarizes postoperative complications and is more sensitive than existing morbidity endpoints, serving as a standardized and widely applicable primary endpoint in research [33]. In addition, the total duration of mechanical ventilation, ICU, and total hospitalization length of stay were significantly reduced. In this same line, patients in the intervention group presented lower rates of critical patient myopathy (defined as a clinical picture of exaggerated diffuse muscle weakness, paresis, and dysphagia, with failure to wean from mechanical ventilation, the need for intensive physiotherapy to recover, and/or accelerated reduction of corticoid therapy) and paralytic ileus, also observed in a previous investigation in patients undergoing major abdominal surgery [16], potentially suggesting that prehabilitation could aid early mobilization after surgery. Besides, the reduction of discharged patients to rehabilitation facilities in the prehabilitation group points out a possible reduction of disability after heart transplantation, as previous literature suggests [19,53].

Although we did not monitor the impact of nutritional and psychological interventions with an objective measure, presumably both would have influenced functional capacity improvement. Nutritional intervention presumably helped to ensure enough substrate to take profit from the optimal effects of exercise [13], and the psychologic support with mindfulness-based therapy helped to boost and maintain a positive patient attitude. As already demonstrated in other settings [16], high adherence to the program and its efficacy may have been favored by the positive physical and mental benefits. A recent review in cardiovascular surgeries emphasizes that the perspective of an upcoming major surgery offers unique opportunities (“teachable moments”) to improve patient attitude by adopting healthy lifestyle habits to optimize clinical outcomes [17].

In elective surgery, the reported length of the prehabilitation programs ranges from 3 to 6 weeks in cancer abdominal surgery and from 2 to 10 weeks in cardiac surgery. However, heart transplantation candidates who are malnourished, sarcopenic, and/or frail might need longer training periods. In this sense, it is important to note that the long-term sustainability of the beneficial effects of prehabilitation on physical status has not been evaluated. Our study evaluated the effects of prehabilitation immediately following the intensive phase (8 weeks), not just before the heart transplantation. In the heart transplantation context, the patient may be waiting for several months, and thereby surgery can occur after a significant interval has elapsed following the end of the structured program. Presumably, the benefits of prehabilitation on postoperative outcomes can be highly dependent on the development of enduring health habits that persist until transplantation and into the post-transplant phase. Based on this assumption, we decided to extend the program (maintenance phase) to reinforce these habits and maintain the observed benefits over time until transplantation.

The impact of exercise training on healthcare use and medical costs in chronically stable patients has been widely assessed within the context of cardiopulmonary rehabilitation programs but never investigated in the setting of pre-transplant patients. Similarly to our previous study in major abdominal surgery [58], the economic analysis of the current investigation showed that introducing prehabilitation adds cost to the heart transplantation procedure, but this over-cost seems to be offset by the reduction of complications and hospital stay. 

Cost/value analysis should support clinical recommendations in an era of increasing healthcare costs in heart failure but remains infrequent [59]. Approximately 75–80% of the direct costs for heart failure are attributable to inpatient hospital stays but are also related to more discharges to long-term care facilities [60]. Unfortunately, our study did not assess indirect (societal) costs and it was not designed with the statistical power to prove the potential cost-saving effect of prehabilitation thus preventing the generalization of the results.

Whereas the benefit of the multimodal prehabilitation program on postoperative outcomes seems to be clear, the precise mechanism underlying, and the degree to which preoperative modification of such factors (i.e., functional capacity (VO_2_), frailty score, nutritional status, etc.) affect postoperative outcome have not been clarified. The aim of our study was not to elucidate this; hence, some aspects were not reassessed after the program. The authors hypothesize that the benefits of prehabilitation are mainly attributable to the already established enhancement of functional capacity; however, the improvement of frailty, sarcopenia, or nutritional status, which has not been assessed, would also contribute to explaining the benefits of the program.

We recognize the limitations as it is a single-center design, small sample size, potential for recruitment bias, difficulties in comparing with a retrospective no-intervention cohort, and the generalization of its findings. As a non-randomized study, our results are subject to confounding and potential patient selection bias. We addressed this by including many candidates in the control group, including those patients admitted to the waiting list since January 2014 and undergoing heart transplantation in a 7-year period, when there have been no relevant changes neither in the surgical technique nor in the clinical management, so treatment and standard of care have been similar in both groups. Besides, a randomized control trial design in this population may be ethically debatable regarding the harmful effects of physical inactivity during the weeks or even months of the waiting list period. In fact, the groups were clinically comparable, the only remarkable difference was the time on the waiting list, significantly longer in the prehabilitation group. This may be explained in part by the COVID-19 pandemic situation (time on the waiting list was influenced (enlarged) by the COVID-19 situation).

Given the study design, it lacks frailty assessment in the control group, and hypothetically the experimental group may have been in better physical shape or “less frail” causing improved outcomes. However, we might assume that both groups are comparable since other aspects that characterize the frail status (i.e., functional capacity, exercise tolerance, age, prognostic score, etc.) were not different.

## 5. Conclusions

In summary, the current study points out that personalized multimodal prehabilitation in advanced heart failure patients awaiting heart transplantation is safe, favorably impacts the short-term postoperative outcome and it is likely cost-effective. Although further multicenter, larger, and cost-benefit analyses are needed to strengthen evidence and assess limitations of the scalability of prehabilitation programs, the heart transplantation waitlist period takes place in a setting with substantial opportunities to rationalize and redesign pathways of care towards this population benefit. Prehabilitation programs offer the opportunity to go beyond the traditional “waiting-list status” and shift to an “active waiting-list status”, thereby improving the patient’s condition before and after heart transplantation. Prehabilitation would also offer the possibility of long-term behavioral changes with the consequent improvement in long-term survival and quality of life.

## Figures and Tables

**Table 1:** Demographics and Baseline Clinical Characteristics.

	Control Group (N = 51)	preHAB Group (N = 31)	*p*-Value
Age (years)	56 ± 12	54 ± 13	0.580
Male n (%)	30 (59)	25 (81)	0.072
Smoker Status n (%)			0.346
Former Smoker	27 (53)	21 (68)	
End-Stage Heart Failure Etiology n (%)			0.639
Ischemic cardiomyopathy	18 (35)	13 (42)	
Dilated cardiomyopathy	17 (33)	8 (26)	
Hypertrophic cardiomyopathy	6 (12)	5 (16)	
Amyloid cardiomyopathy	1 (2)	2 (7)	
Toxic	2 (4)	0 (0)	
Others	7 (14)	3 (10)	
Charlson Comorbidity Index	3.00 (2–5)	4.00 (3–6)	0.376
Obesity (IMC >30) n (%)	8 (16)	7 (23)	0.625
Previous cardiac surgery n (%)	13 (26)	10 (32)	0.683
Levosimendan chronic treatment	14 (28)	13 (42)	0.267
Pulmonary hypertension			0.911
Moderate n (%) (PSAP 40–60 mmHg)	13 (26)	7 (23)	
Severe n (%) (PSAP > 60 mmHg)	15 (29)	10 (32)	
Specific pulmonary hypertension treatment n (%)	16 (31)	11 (36)	0.887
Bosentan	8 (16)	8 (26)	0.404
Sildenafil	10 (20)	6 (19)	1.000
Right heart catheterization			
Cardiac index (L/min/m^2^)	2.4 ± 1	2.2 ± 6	0.190
Pulmonary vascular resistance (Wood units)	1.9 ± 1	2.6 ± 2	0.014
Left ventricle ejection fraction %	25 (20–29)	22 (20–35)	0.693
Arrhythmia history n (%)			
No previous arrhythmia history	10 (20)	11 (36)	0.181
Atrial fibrillation	31 (61)	16 (52)	0.559
Cardiac resynchronization therapy	9 (18)	5 (16)	1.000
Implantable cardioverter- defibrillator	41 (80)	25 (81)	1.000
CFS-CSHA	Not available	4 [4,5]	N/A
Frailty (CFS-CSHA ≥5)	Not available	13 (42)	N/A
INTERMACS (Last month previous to HT)			0.080
3	4 (8)	1 (3)	
4	17 (34)	9 (29)	
5	20 (40)	14 (45)	
6	9 (18)	3 (10)	
7	0 (0.0)	4 (13)	
Time in waiting list (days)	100 (28–277)	186 (93–368)	0.016

Data are presented as means ± SDs, N (%), and medians (Q1–Q3) appropriately. Abbreviations: SD, standard deviation; NYHA, New York Heart Association; CFS-CSHA, Clinical Frailty Score from the Canadian Study of Health and Aging; INTERMACS, Interagency Registry for Mechanically Assisted Circulatory Support; HT, heart transplantation.

**Table 2:** Impact of prehabilitation program on functional capacity, physical activity, hand grip, psychological status, and quality of life.

	Before Intervention	Post-Program	*p*-Value
CPET parameters			
Peak oxygen pulse (VO_2_/HR) (mL/beats)	8 (5.92–10.05)	10.4 (8.35–11.1)	0.01
Ventilatory efficiency (VE/VCO_2_) at anaerobic threshold	39.06 ± 6.19	35.85 ± 6.11	0.077
Oxygen uptake at anaerobic threshold (AT VO_2_) (mL/kg/min)	7.91 ± 2.22	9.25 ± 1.94	0.033
Peak oxygen uptake (VO_2_max) (mL/kg/min)	10.1 (8–13)	12.5 (10 -14.78)	0.034
Peak oxygen uptake (VO_2_max) % ref	33 (25–45)	42 (33–50)	0.026
Peak work-rate (watts)	66.85 ± 27.56	85.67 ± 30.20	0.013
Endurance time (seconds)	281 (208–380)	728 (397–900)	<0.001
6MWT (meters)	411 (355.5–490.5)	453 (424–514.3)	0.081
Sit-to-stand (repetitions)	10 ± 4	14 ± 6	0.013
YPAS total	24 (15–37)	49 (38–60)	<0.001
Hand grip dominant hand (kg)	33 ± 10	37 ± 10	0.248
Hand grip non-dominant hand (kg)	31 ± 10	33 ± 9	0.518
HADS-anxiety	5 (3–8)	4 (2–7)	0.34
HADS-depression	4 (2–7)	4 (3–7)	0.87
MLHFQ	58 ± 19	47 ± 19	0.046

Data are presented as means ± SDs, N (%), and medians (Q1–Q3) appropriately. Abbreviations: SD, standard deviation; YPAS, Yale Physical Activity Score; 6MWT, 6-min walking test; HADS, Hospital Anxiety and Depression Scale; MLHFQ, Minnesota Living with Heart Failure Questionnaire.

**Table 3:** Postoperative Outcomes.

	Control Group (N = 51)	preHAB Group (N = 31)	*p*-Value
Total number of complications per patient	5 (3–8)	3 (2–3)	<0.001
Minor complications (Clavien-Dindo 1 or 2) per patient	4 (3–6)	2 (1–3)	<0.001
Major complications (Clavien-Dindo ≥3) per patient	0 (0–2)	0 (0–1)	0.242
Medical complications	5 (3–7)	2 (1–3)	<0.001
Surgical complications	0 (0–1)	0 (0–1)	0.167
Comprehensive Complication Index (CCI)	37 (30–72)	31 (23–41)	0.033
Mechanical ventilation time (hours)	37 (12–143)	20 (12, 52)	0.032
ICU length of stay (days)	7 (5–14)	5 (3, 7)	0.010
Primary graft failure (%)	5 (10)	1 (3)	0.502
Surgical reinterventions during HT hospitalization	7 (14)	5 (16)	1.000
Hospitalization length of stay (days)	23 (18–38)	18 (16–22)	0.008
Discharge destination (%)			0.009
Home	33 (65)	29 (94)	
Nursing/rehabilitation facilities	16 (31)	1 (3)	
In-hospital mortality	2 (4)	1 (3)	1.000
30-days after HT mortality	1 (2)	0 (0)	1.000
3-months after HT mortality	1 (2)	2 (7)	0.657
1-year after HT mortality	3 (6)	3 (10)	0.839

Data are presented as means ± SDs, N (%), and medians (Q1–Q3) appropriately. Abbreviations: CCI, Comprehensive Complication Index; ICU, Intensive Care Unit; HT, heart transplantation; SD, standard deviation.

**Table 4:** Specified/Disaggregated complications.

	Control Group (N = 51)	preHAB Group (N = 31)	*p*-Value
Arrhythmia requiring antiarrhythmic drugs	15 (29)	1 (3)	0.009
Arrhythmia requiring electrical cardioversion	3 (6)	0 (0)	0.442
Myocardial infarction	1 (2)	0 (0)	1.000
Cardiac arrest	1 (2)	1 (3)	1.000
Primary graft failure	5 (10)	1 (3)	0.502
ECMO/LVAD	6 (12)	1 (3)	0.350
Respiratory insufficiency requiring NIV/HFNC	5 (10)	0 (0)	0.186
Respiratory insufficiency requiring intubation	2 (4)	2 (7)	1.000
Difficult weaning/tracheostomy	2 (4)	2 (7)	1.000
Respiratory tract infection	14 (28)	3 (10)	0.100
Pleural effusion requiring chest-tube placement	5 (10)	1 (3)	0.502
Critical patient myopathy requiring intensified rehabilitation	15 (29)	2 (7)	0.027
Acute kidney injury requiring furosemide perfusion	26 (51)	10 (32)	0.154
Acute kidney injury requiring kidney replacement therapy	9 (18)	6 (19)	1.000
Nausea/vomiting	20 (39)	7 (23)	0.190
Paralytic ileus	10 (20)	0 (0)	0.022
Hyperglycemia requiring insulin infusion	22 (43)	8 (26)	0.179
Pressure ulcers	3 (6)	0 (0)	0.442
Digestive hemorrhage	6 (12)	0 (0)	0.122
Delirium	16 (31)	5 (16)	0.203
Deep venous thrombosis	4 (8)	3 (10)	1.000
Pulmonary thromboembolism	0 (0)	0 (0)	not applicable
Stroke	1 (2)	1 (3)	1.000
Urinary tract infection	3 (6)	0 (0)	0.442
Catheter-related bloodstream infection	7 (14)	2 (7)	0.511
Other infections	14 (28)	5 (16)	0.364
Reintervention	7 (14)	5 (16)	1.000
Post-surgical hemorrhage	6 (12)	2 (7)	0.687
Surgical site infection	4 (8)	1 (3)	0.710
Cardiac effusion/cardiac tamponade requiring drainage	4 (8)	5 (16)	0.424
Pneumothorax/hemothorax	5 (10)	2 (7)	0.905

Data are presented as means ± SDs, N (%), and medians (Q1–Q3) appropriately. Abbreviations: ECMO/LVAD, ExtraCorporeal Membrane Oxygenation/Left Ventricular Assist Device; NIV/HFNC, Non-Invasive Ventilation/High-Flow Nasal Cannula.

**Table 5:** Descriptive cost statistics.

Group	N	Median	1st Quartile	3rd Quartile	*p*-Value
Prehab	31	49,771 €	44,999 €	54,432 €	0.254
Standard Care	51	54,748 €	45,765 €	79,777 €	

*p*-value: calculated through quantile regression; non significant at 90%.

## Data Availability

All data is contained within the current article. The data presented in this study are available on request from the corresponding author.

## Supplementary Materials

The following supporting information can be downloaded at: https://www.mdpi.com/article/10.3390/jcm12113724/s1. Table S1. Baseline characteristics from the baseline assessment performed on 46 patients that initially consented to participate (extended group).
