# Supplementary material for: Multimodal Prehabilitation in Heart Transplant Recipients Improves Short-Term Post-Transplant Outcomes without Increasing Costs
Source: J Clin Med. 2023 May 28;12(11):3724. doi: 10.3390/jcm12113724 (PMC10253351; doi:10.3390/jcm12113724)
Supplement: Supplementary file 1 [file jcm-12-03724-s001.zip › jcm-2386636-supplementary.pdf]

Supplementary material

Table S1. Baseline characteristics from the baseline assessment performed on 46 patients that initially consented to participate (extended group).

	Prehab Baseline assessment (N=46)
Age (years)	54 ± 14
Male	30 (80)
Transplantation type= Re-transplantation	2 (4.3)
Smoker Status	
Former Smoker	43 (93.4)
End-Stage Heart Failure Etiology	
Ischemic cardiomyopathy	19 (41.3)
Dilated cardiomyopathy	12 (26.1)
Hypertrophic cardiomyopathy	6 (13)
Amyloid cardiomyopathy	2 (4.3)
Others	6 (13)
Time in heart transplantation waiting list (days)	247 (21 - 735)
Charlson Comorbidity Index	4 (1 - 9)
CFS-CSHA	4 (4 - 5)
Frailty (CFS-CSHA ≥5)	16 (40)
Arterial hypertension	20 (43.4)
Dyslipidemia	20 (43.4)
Diabetes Mellitus	14 (30.4)
Arterial peripheral disease	7 (15.2)
Ischemic heart disease	20 (43.4)
Hypothyroidism	5 (10.8)
Obstructive sleep apnea	5 (10.8)
Stroke	7 (15.2)
COPD/Asthma	10 (21.7)
Chronic kidney disease	18 (39.1)
Obesity (IMC >30) n (%)	9 (19.5)
Previous cardiac surgery	14 (30.4)
Levosimendan	17 (36)
Pulmonary hypertension	
No	12 (26)
Mild	8 (17.4)
Moderate	11 (23.9)
Severe	14 (30.4)
Pulmonary hypertension treatment	15 (32.6)
Bosentan	12 (26.1)
Sildenafil	8 (17.4)
Left ventricle ejection fraction %	21 (12 - 65)
Arrhythmia history	
No previous arrhythmia history	14 (30.4)
Atrial fibrillation	25 (54.3)
Auriculoventricular block	2 (4.3)
Other arrhythmias	11 (23.9)

**Pacemaker**	5 (16.1)
**Implantable cardioverter-defibrillator**	35 (76.1)
**INTERMACS (last month previous to HT)**	
**3**	2 (4.3)
**4**	14 (30.4)
**5**	14 (30.4)
**6**	6 (13)
**7**	7 (15.2)

Data are presented as means ± SDs, N (%), and medians (Q1–Q3) appropriately. Abbreviations: NYHA, New York Heart Association; INTERMACS, Interagency Registry for Mechanically Assisted Circulatory Support; COPD, Chronic obstructive pulmonary disease.
